# Motivation for feedback-seeking among pediatric residents: a mixed methods study

**DOI:** 10.1186/s12909-018-1253-8

**Published:** 2018-06-19

**Authors:** Duncan Henry, Travis Vesel, Christy Boscardin, Sandrijn van Schaik

**Affiliations:** 1 Department of Pediatrics, University of California San Francisco, 550 16th Street, 5th Floor San Francisco, San Francisco, CA 94143 USA; 2 Department of Pediatrics, Duke University, San Francisco, USA; 3 Department of Medicine, University of California San Francisco, San Francisco, USA

**Keywords:** Feedback, Feedback-seeking, Intrinsic motivation, Self determination theory

## Abstract

**Background:**

For effective self-directed life-long learning physicians need to engage in feedback-seeking, which means fostering such behavior during training. Self-determination theory (SDT) posits that intrinsic motivation is fostered when the environment optimizes the individual’s experience of autonomy, relatedness, and competence. Educational settings meeting these psychological needs may foster intrinsic motivation in trainees, enhance their desire for feedback, and promote feedback-seeking. We sought to examine residents’ feedback-seeking behaviors through the lens of SDT and explore the association with intrinsic motivation and career choice.

**Methods:**

We used a mixed-methods approach with an explanatory sequential design. Residents participated in simulation training, completed an inventory of intrinsic motivation (IMI) and responded to sequential opportunities for performance feedback requiring different levels of effort. We compared IMI scores and career choice between groups with different effort. We interviewed high-effort feedback-seekers and conducted thematic analysis of interview data.

**Results:**

Thirty-four of 35 residents completed the survey (97%). Of those completing the study, 12 engaged in low-effort feedback-seeking only, 10 indicated intent for high-effort feedback-seeking and 10 actually engaged in higher effort to get feedback. Groups did not differ in mean IMI scores. Among high-effort feedback-seekers more residents were interested in critical care–related fields compared to the other groups.

We identified 5 themes around autonomy, relatedness, and competence clarifying residents’ reasons for feedback-seeking.

**Conclusions:**

Our findings suggest that among residents, the relationship between motivation and feedback-seeking is complex and cannot be predicted by IMI score. Career plans and relationships with feedback providers impact feedback-seeking, which can inform educational interventions.

**Electronic supplementary material:**

The online version of this article (10.1186/s12909-018-1253-8) contains supplementary material, which is available to authorized users.

## Background

Feedback is thought to be essential for skill development and performance enhancement in medical education, but little is known about what motivates medical trainees to seek feedback [1–3]. Archer proposed a model for effective feedback in the health professions in which feedback is part of a learner-driven process of reflection built on self-monitoring. Since self-assessment is frequently inaccurate, external feedback is essential for self-monitoring [4, 5]. This insight has led to the conceptualization of informed self-assessment, defined as “the set of processes through which individuals use external and internal data to generate an appraisal of their own ability.” [6] Informed self-assessment and the associated process of feedback-seeking is thought to be the cornerstone of self-directed, life-long learning, a requirement of all physicians [4, 7]. While undergraduate and graduate medical programs are required to routinely provide trainees with performance feedback; after completion of training, opportunities for structured feedback become rare [8]. Hence, trainees should start to engage in feedback-seeking during medical training.

Published data suggest medical trainees feel they do not get sufficient structured feedback, but to what degree they compensate by seeking out feedback themselves is less clear [9–12]. The limited available data on feedback-seeking is based on self-report and suggests a few factors that promote feedback-seeking in medical trainees. Feedback-seeking is more common among high-achieving individuals, if feedback is likely to lead to affirmation or have a positive impact on self-image, or if trainees perceive their attending’s supervisory style as supportive [13–17]. Whether self-reported feedback-seeking matches actual behaviors and what promotes such behaviors has received limited attention to date. Self-determination theory (SDT) may provide a useful lens through which to view feedback-seeking behavior and the variables that influence it. SDT posits that in order to encourage intrinsic motivation (defined as free engagement in an activity out of interest or inherent satisfaction) learning environments should promote three characteristics: autonomy, competency, and relatedness [18–20]. Autonomy refers to the ability of the individual to choose what they consider a useful course of action. It is supported by creating an environment where learners are empowered to set their own goals and direct their learning. Competency is embodied in the desire to feel effective in action and performance. Relatedness refers to a sense of interconnectedness, belonging, and engagement in reciprocal caring relationships, for example incorporation of the learner into the larger professional or clinical group [21]. Educational environments that meet these psychological needs may foster intrinsic motivation in trainees, enhance their desire for feedback, and thus promote feedback-seeking.

This study aimed to document residents’ actual feedback-seeking behaviors, determine the association with intrinsic motivation and explore potential factors influencing feedback-seeking behavior. We conducted the study in the educational context of simulated pediatric emergencies, which may have different appeal to residents with different career aspirations, and therefore explicitly examined the impact of career choice on feedback-seeking. The overall goal of our study was to gain information on how educational environments can promote feedback-seeking among learners.

## Methods

### Design

This mixed methods study used an explanatory sequential design complementing data gathered from surveys with interview data [22, 23].

### Participants and setting

We conducted the study in the context of a simulation-based team-training program for pediatric residents and nurses at our institution. This program, described in detail elsewhere [24], consists of bi-monthly 1-h sessions focused on emergency situations with 2 scenarios lasting 10–15 min each followed by similar length, semi-structured, group debriefings led by trained instructors. All residents participating in these sessions as team leaders between August 2013 and July 2014 were eligible for the study, which was approved by the University of California San Francisco (UCSF) Committee on Human Research.

### Feedback opportunities

We created two sequential opportunities for residents to seek further feedback (beyond that offered during the debriefing) which required different degrees of effort allowing for comparison of trainees who invested more effort with those who invested less. The first opportunity involved written feedback on teamwork skills based on video review of the resident’s performance by two study investigators (DH, TV) using the TeamSTEPPS framework [25]. We imported feedback comments into an on-line instrument (SurveyMonkey**™**) and gave interested residents access. Seeking out this feedback required low effort – accessing an email link. The second opportunity consisted of a 30 min, in-person feedback session with the principal investigator (DH). The resident had to take initiative for scheduling this session and make time for it; this therefore required higher effort.

### Instruments

We utilized a previously validated tool, the Intrinsic Motivation Inventory (IMI), a 22-item survey with 4 sub-scores on 7-point rating scales, to assess resident motivation as it relates to participating in the simulation. We made minor adaptations to the tool, replacing generic wording in the original tool indicating “an activity” with words that referenced the simulation activity specifically. The first sub-score, Interest/Enjoyment, is a direct self-reported measure of intrinsic motivation, whereas the other 3, Perceived Choice, Perceived Competence, and Pressure/Tension are indirect measures [26]. (Additional file 1: Appendix A).

Using sensitizing concepts from SDT, we created a semi-structured interview guide to examine factors that influenced residents’ participation in an in-person feedback session, their experience with the feedback, and their overall attitude towards feedback-seeking. (Additional file 2: Appendix B).

### Procedures

**Fig. 1:**
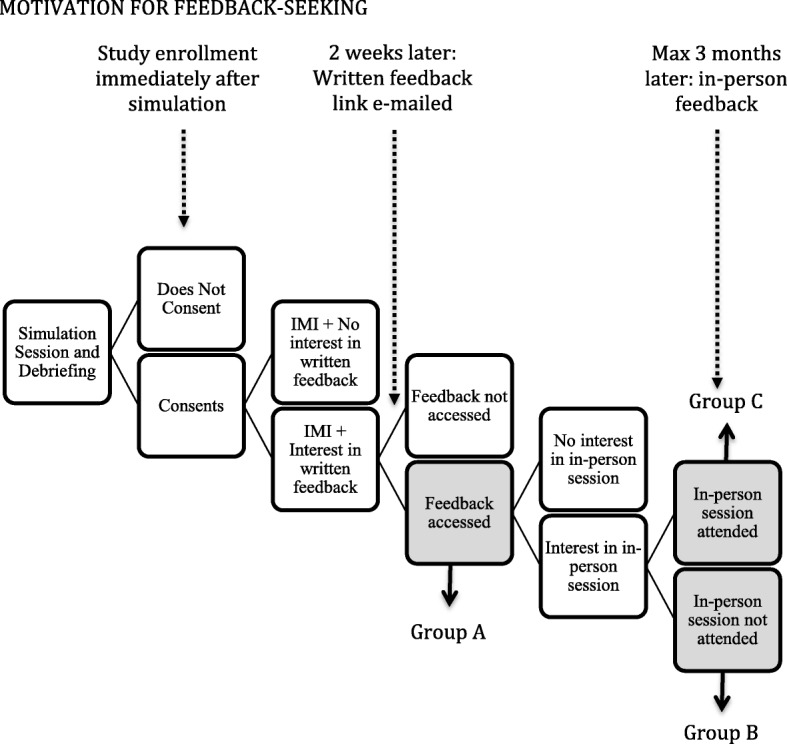
Study procedures. Residents participating as team leaders were recruited to the study immediately after the simulation session, and those who consented completed the IMI and indicated whether they were interested in written feedback. Those interested received feedback via e-mail, with a link to survey questions about career choice and the opportunity to request additional feedback in an in-person session. Those who indicated interest were prompted to e-mail the study investigator with potential dates and times for the session. These study procedures led to 3 study groups: Group A: low effort feedback-seeking; Group B: intended high effort feedback-seeking; Group C: actual high effort feedback-seeking

Figure 1 shows the study design with the different groups based on feedback-seeking opportunities. Immediately following the simulation session, consented residents completed the IMI and indicated their interest in additional performance feedback. Two weeks later, we e-mailed those who indicated interest a link to the written feedback. At the end of the feedback we included questions about their anticipated career choice and their interest in an in-person feedback session. Those who indicated interest were prompted to e-mail the study investigator with potential dates and times for the session. Residents who scheduled an in-person feedback session met individually with the principal investigator (DH) to discuss their performance and subsequently participated in a 30-min semi-structured interview. Interviews were audio-recorded and transcribed. In each phase of the study, residents who indicated interest in additional feedback but did not act on this received one reminder email after 1 month. They were excluded from subsequent phases of the study if they did not pursue their indicated interest (either accessed their feedback or scheduled an in-person session) within 3 months.

### Quantitative analysis

We grouped participants according to their feedback-seeking behaviors as indicated in Fig. 1: Group A, low effort feedback-seeking, Group B, intended high effort feedback-seeking, and Group C, actual high effort feedback-seeking. We calculated descriptive statistics for IMI sub-scores and compared scores between the 3 groups using analysis of variance (ANOVA). To analyze the impact of career choice on feedback-seeking, we grouped residents into two categories: those with high need for skills taught in the simulation sessions (Critical Care-related fields, including Critical Care, Neonatology, and Emergency Medicine) and those with lower need (all other subspecialties, primary care, and undecided). We used Fisher’s exact test to compare proportions of residents in each career group among the three study groups.

We used SPSS 16.0™ for all statistical calculations.

### Qualitative analysis

Two investigators (DH, SvS) independently coded the first three transcripts, compared codes, and generated a preliminary coding tree. Next, two investigators (DH, TV) each coded two additional transcripts using the preliminary coding tree and finalized the coding scheme which they used to code all remaining transcripts. They compared, discussed, and reconciled differences and a separate investigator (SvS) reviewed the analysis for accuracy. We conducted a thematic analysis of the interview data and used a constant comparison approach to identify and verify themes and sub-themes [27, 28]. We used DeDoose**™** to organize and analyze qualitative data.

## Results

### Quantitative results

Of 43 eligible residents, we excluded 8 because of predicted scheduling difficulties for in-person feedback. The remaining 35 residents all consented to the study, completed the IMI and opted to receive written feedback. The response rate was 97% as one resident did not access the written feedback and was excluded from further analysis. Of the remaining 34 residents, 22 (65%) indicated interest in an in-person feedback session, but only 10 (45%) eventually scheduled and attended such a session. Table 1 provides the distribution of residents per study group, and Table 2 summarizes average IMI scores per group. Overall, residents demonstrated moderate levels of intrinsic motivation (average subscale scores between 3.87 and 4.94, 1–7 scale) without significant differences between study groups. Thus, intrinsic motivation, as measured by the IMI, did not differ between residents who engaged in feedback-seeking behaviors requiring different levels of effort. Of the 34 residents included in the analysis, 12 anticipated a career in a critical care-related field, while 22 did not. The group engaged in high effort feedback-seeking contained a higher proportion of residents interested in critical care–related fields compared to the two groups with lower effort feedback-seeking (7/10 in group C, versus 3/12 in group B and 2/12 in group A; *P* = 0.03; Fisher’s Exact test).

**Table 1:** Study group description

Study Group	Number in analysis	Comments
Group A: low effort feedback-seeking; written feedback only	*n* = 12	One resident indicated interest but never accessed written feedback, was excluded from analysis
Group B: intended high effort feedback-seeking; no in-person session scheduled/attended	n = 12	Never sent an email request (*N* = 5) Sent email request but did not respond to follow-up emails (N = 2) Unable to schedule a working time for session (*N* = 3) Scheduled but did not show up (*N* = 2)
Group C actual high effort feedback-seeking; in person session scheduled and attended	*n* = 10

**Table 2:** Mean IMI scores by study group based on feedback seeking behaviors

IMI Sub-Score	All Participants (*n* = 34)	Group A Low effort (*n* = 12)	Group B Intent to high effort (*n* = 12)	Group C Actual high effort (*n* = 10)
Interest Enjoyment	4.83 ± 0.97	4.52 ± 0.68	4.79 ± 0.99	5.26 ± 1.16
Perceived Choice	4.38 ± 1.58	3.78 ± 1.71	4.60 ± 1.47	4.82 ± 1.48
Perceived Competence	3.85 ± 1.18	3.83 ± 1.28	3.87 ± 1.13	3.84 ± 1.24
Pressure Tension	4.95 ± 0.96	5.25 ± 0.71	4.68 ± 1.05	4.92 ± 1.09

Values represent means ± standard deviation. We found no significant differences between groups on one way ANOVA (*p* > 0.2 for all comparisons)

### Qualitative results

Through analysis of interview data collected among residents who participated in the in-person session we identified 5 themes that clarified their reasons to participate in seeking additional feedback, their experience with the session and their overall experience with feedback. We grouped the themes into three domains mirroring our theoretical underpinnings from SDT: autonomy, relatedness, and competence. Within the domain of autonomy, we identified two themes: “Having a conversation” and “How I learn.” Residents in our study wanted feedback from someone with whom they could engage in a dialogue, processing information in a manner that was meaningful for their own learning. Within the domain of relatedness, we recognized two additional themes: “Importance of trust” and “Importance of shared goals.” Residents reported seeking feedback from someone they trusted and who was likely to share their individualized goals. Finally, in regards to the domain of competence, we isolated the theme: “Improving patient care skills”. We found that residents sought feedback aimed at improving skills they anticipated needing to provide patient care in the future. Table 3 details the themes with representative quotes.

**Table 3:** Major themes and representative quotes from interviews with residents participating in in-person feedback

*Domain: Autonomy*	
* Theme 1: Having a conversation.* Residents identified that the ability to engage the feedback provider in conversation and discuss feedback content was a key mediator of feedback-seeking.	I guess for this, since it was an option, it’d be really great to get feedback, to build more skills, and to be able to ask questions (R15) I had specific kinds of concerns about areas of my performance that I wasn’t sure exactly how to improve on in the future, and we talked about those and discussed strategies for improving them. (R21)
* Theme 2: How I learn* Residents sought feedback when they perceived the feedback environment to be complimentary to how they learned best.	I’m the kind of person that does need to process (…) if I’m one person, and it’s like a one-on-one teaching session, then I can do that process with one person, (….) and basically control the traffic of the information exchange (R18) I felt like I knew enough of the medicine that I could kind of reassure myself but it’s definitely not as helpful just to think about it on my own as to get feedback from someone else who’s more experienced (R21)
*Domain: Relatedness*	
* Theme 3: Importance of trust.* Trainees are more likely to engage in feedback-seeking if they trust the feedback source.	You’re someone I trust and look up to, and I know you’re very reasonable. So, when you write feedback, if you say something good, I will believe it, and if you say something bad, I will believe it too. It’s just more acceptable to me (R18) Yeah, there’s certain things like just knowing (….) and also feeling comfortable with you maybe encouraged me to do it. (R26)
* Theme 4: Importance of shared goals* Trainees seek feedback when they perceive that feedback providers share their developmental goals	I think it’s really helpful … it helps that I’ve worked with you before, that I know what your goals are, that they’re the same goals that I have. (R15) Those are, pieces that I expect you to comment on … the things that you’re valuing and the things that I’m expecting you to value. (R18)
*Domain: Competence*	
* Theme 5: Improving patient care skills*	(…) because I know what my future is, and if there’s a real code in three months from now and I’m a fellow, I don’t want to not have done something because I was too wimpy to sit down for feedback. (R18) I think that you can’t, especially in emergency situations, you can’t have too much feedback about those. I think it’s one of the most important skills we have to develop, especially for people who are interested in going into more critical care type of things (R5)

## Discussion

In our study of residents’ feedback-seeking behavior, the majority of residents engaged in feedback-seeking if it required limited effort, but relatively few sought feedback if it required increased effort. We found no association between intrinsic motivation (as measured by the IMI) and feedback seeking behaviors, while there was an association with anticipated career choice: residents interested in critical-care and related fields were more likely to engage in high effort feedback-seeking. While not evident from the quantitative data, our qualitative data offered evidence that factors related to intrinsic motivation contribute to feedback seeking.

Our inability to detect a significant relationship between IMI scores and feedback-seeking has several possible explanations. First, our sample size was small and limited the power to detect significant differences. Second, our hypothesis may simply have been incorrect and feedback-seeking behavior in our educational context is not intrinsically motivated, at least not in a way that is measured by the IMI instrument. This instrument measures motivation as it relates to the simulation experience, which may not represent the focus of intrinsic motivation that drove residents in our study to feedback-seeking.

Our qualitative data however suggest that some form of intrinsic motivation does play a role, with themes that mapped to the different domains of self-determination theory: autonomy, relatedness, and competence. Autonomy manifested in trainees’ desire to engage in a conversation they could direct, relatedness in their discussion of trust and shared goals with the feedback provider, and competence in their focus on accomplishing short and long-term personal goals. These findings are in accordance with prior studies examining self-identified drivers of feedback-seeking behavior among medical trainees. These include perceiving a feedback providing supervisor as supportive, the desire to ask questions, and to learn from feedback [16, 29, 30]. Moreover, our data also resonate with a recent study published by Ramani et al., which highlighted that trainees place value on receiving feedback in the context of a dialogue intended to promote mutual professional growth [31]. In that study residents voiced the importance of corrective feedback, as did the residents in our current study as well as in a prior study in the pediatric residency simulation setting [32]. This finding is consistent with published data on what type of feedback interests residents [33], and of particular interest in light of self-determination theory because corrective feedback can collide with feelings of competence [34]. Apparently these residents overcome this conflict if they feel they can gain critical skills or patient outcomes are at stake.

Overall, the qualitative data paint a clear picture of what motivated residents in our study to engage in high-effort feedback-seeking and what they valued in the experience: they were interested in a conversation with someone they trusted, shared their goals, and focused on developing skills that were relevant to them. It is therefore not too surprising that career choice was associated with resident’s feedback-seeking behavior. The in-person session put residents in direct conversation with someone in their intended career direction, who therefore has similar goals and skills these residents want to develop. Thus, this in-person session provided an opportunity to establish an “educational alliance”, a relationship with mutual focus on improving performance and professional capabilities [35, 36]. Our data suggest the residents in our study had a goal-oriented approach to feedback-seeking rather than a performance-oriented approach; the former appears to be associated with higher perceived benefits of feedback and thus may enhance feedback seeking [16]. Residents may not have viewed the in-person session as a mechanism to get specific performance feedback related to the simulation, but rather as a chance to work towards goals by connecting with a potential coach. Coaching is gaining traction in medicine because of its specific focus on continuous skill improvement [37, 38]. Aligning opportunities for residents to engage in feedback-seeking with coaching may be an effective means of promoting skills essential for self-directed, life-long learning.

In addition to limited power due to small sample size, our study has other limitations. First, we did not interview residents who chose not to engage in further feedback-seeking behavior and therefore have limited insights into their motivating factors. Second, only one person (DH) conducted the in-person feedback sessions, which could have affected residents’ interest if they lacked a sense of relatedness to this individual. However, only after a “Yes” response did the survey indicate with whom the in-person session would take place. Finally, our study was limited to feedback-seeking behavior in one context (simulated team training) among a single group of learners (pediatric residents) at one institution which may limit generalizability of our findings. We view these limitations as opportunities for additional research, and future studies should examine professional goals and the learning context more carefully to determine how best to reinforce feedback-seeking for a variety of trainees across contexts and disciplines.

## Conclusion

The relationship between motivation and feedback-seeking is complex. Providing more opportunities for feedback does not universally increase feedback-seeking and the interaction between career goals and certain elements of self-determination, in particular relatedness and competence, may be more important. At the residency level, seeking context-specific performance feedback may be less relevant than establishing longitudinal professional relationships that encompass aspects of coaching. As trainees are expected to become self-directed learners skilled at informed self-assessment, our educational programs should reflect these priorities. If systems are put in place that offer trainees opportunities to seek feedback from people who can serve as coaches in light of their career aspirations, they may be more likely to actually pursue and value such opportunities for feedback which in turn may promote future feedback-seeking.

## Additional files

Additional file 1: **Appendix A.** Intrinsic Motivation Inventory. Includes the intrinsic motivation inventory (IMI) which we adapted to the specific activity of running a mock code. Information on how to calculate sub-scores for the IMI is included at the end of the inventory. (DOCX 114 kb)

Additional file 2: **Appendix B.** Interview Guide. Includes our interview guide with questions designed to explore participants’ general experience with feedback as well as investigate constructs related to Self-Determination Theory. (DOCX 85 kb)

## Abbreviations

IMI: Intrinsic Motivation Inventory
SDT: Self-Determination Theory
