# Supplementary material for: Motivation for feedback-seeking among pediatric residents: a mixed methods study
Source: BMC Med Educ. 2018 Jun 19;18:145. doi: 10.1186/s12909-018-1253-8 (PMC6007008; doi:10.1186/s12909-018-1253-8)
Supplement: Supplementary file 1 — Appendix A. Intrinsic Motivation Inventory. Includes the intrinsic motivation inventory (IMI) which we adapted to the specific activity of running a mock code. Information on how to calculate sub-scores for the IMI is included at the end of the inventory. (DOCX 114 kb) [file 12909_2018_1253_MOESM1_ESM.docx]

**Appendix A.** Intrinsic Motivation Inventory

**Instructions:** For each of the following 22 statements, please indicate how true it is for you. Please circle only one number (i.e. do not circle both 4 and 5 to indicate a 4.5).

All answers are reported on the following scale:

| 1  Not at all true | 2 | 3 | 4  Somewhat true | 5 | 6 | 7  Very  true |
| --- | --- | --- | --- | --- | --- | --- |
| 1. While I was running the mock code I was thinking about how much I enjoyed it. | | | | | | |
| 2. I did not feel at all nervous about running the mock code. | | | | | | |
| 3. I felt that it was my choice to run the mock code. | | | | | | |
| 4. I think I was pretty good at running the mock code. | | | | | | |
| 5. I found running the mock code very interesting. | | | | | | |
| 6. I felt tense while running the mock code. | | | | | | |
| 7. I think I did pretty well at running the mock code, compared to other residents. | | | | | | |
| 8. Running the mock code was fun. | | | | | | |
| 9. I felt relaxed while running the mock code. | | | | | | |
| 10. I enjoyed running the mock code very much. | | | | | | |
| 11. I didn’t really have a choice about running the mock code. | | | | | | |
| 12. I am satisfied with my performance in running the mock code. | | | | | | |
| 13. I was anxious while running the mock code. | | | | | | |
| 14. I thought running the mock code was very boring. | | | | | | |
| 15. I felt like I was doing what I wanted to do while I was running the mock code. | | | | | | |
| 16. I felt pretty skilled at running the mock code. | | | | | | |
| 17. I thought running the mock code was very interesting. | | | | | | |
| 18. I felt pressured while running the mock code. | | | | | | |
| 19. I felt like I had to run the mock code. | | | | | | |
| 20. I would describe running the mock code as very enjoyable. | | | | | | |
| 21. I ran the mock code because I had no choice. | | | | | | |
| 22. After working at running the mock code for a while, I felt pretty competent. | | | | | | |

^a^The survey creates 4 subscores by averaging questions as follows: Interest/Enjoyment (1, 5, 8, 10, 14R, 17, 20), Perceived Competence (4, 7, 12, 16, 22 ), Perceived Choice (3, 11R, 15, 19R, 21R), Pressure/Tension (2R, 6, 9R, 13, 18). R indicates reversal of the score. (i.e. 1=7)
