# Supplementary material for: Motivation for feedback-seeking among pediatric residents: a mixed methods study
Source: BMC Med Educ. 2018 Jun 19;18:145. doi: 10.1186/s12909-018-1253-8 (PMC6007008; doi:10.1186/s12909-018-1253-8)
Supplement: Supplementary file 2 — Appendix B. Interview Guide. Includes our interview guide with questions designed to explore participants’ general experience with feedback as well as investigate constructs related to Self-Determination Theory. (DOCX 85 kb) [file 12909_2018_1253_MOESM2_ESM.docx]

**Appendix B.** Interview Guide

1. What did you think about the feedback session?

- What did you like most? Why?

- What did you like least? Why?

2. What did you hope to get out of this session? Why did you have those hopes?

3. Did anything surprise you about this session? Why?

4. Did anything upset or frustrate you about this session? Why?

5. What are you going to do with this feedback?

6. In general, how do you learn best?

-What kind of environments? Prompts: With others, alone, on-the-job, reading

- What kind of resources help you? Prompts: People (teachers, peers), technology (internet, video review)

- What kind of instruction? Prompts: Hands-on, case-based

- What kind of feedback?

7. Think back on the written feedback you received, and the session we just had. How do you think they compare? Prompts: in regards to Content/Benefit/Comfort/Limitations/Timing

8. How does this type of feedback session fit into how you learn best?

- Are there other types of contexts where you would use this type of session?

9. When would this type of feedback session not be helpful?

10. Would you do this again? Why?

11. Is there anything else you would like to say to me about feedback in general, or this session in particular?
